# Do patients with very few brain metastases from breast cancer benefit from whole-brain radiotherapy in addition to radiosurgery?

**DOI:** 10.1186/s13014-014-0267-6

**Published:** 2014-12-04

**Authors:** Dirk Rades, Stefan Huttenlocher, Dagmar Hornung, Oliver Blanck, Steven E Schild, Dorothea Fischer

**Affiliations:** Department of Radiation Oncology, University of Lübeck, Ratzeburger Allee 160, Lübeck, 23538 Germany; Department of Radiation Oncology, University Medical Center Eppendorf, Hamburg, Germany; CyberKnife Centre Northern Germany, Güstrow, Germany; Department of Radiation Oncology, Mayo Clinic, Scottsdale, AZ USA; Department of Gynecology and Obstetics, University of Lübeck, Lübeck, Germany

**Keywords:** Breast cancer, Brain metastases, Radiosurgery, Whole-brain radiotherapy, Freedom from new brain metastases

## Abstract

**Background:**

An important issue in palliative radiation oncology is the whether whole-brain radiotherapy should be added to radiosurgery when treating a limited number of brain metastases. To optimize personalized treatment of cancer patients with brain metastases, the value of whole-brain radiotherapy should be described separately for each tumor entity. This study investigated the role of whole-brain radiotherapy added to radiosurgery in breast cancer patients.

**Methods:**

Fifty-eight patients with 1–3 brain metastases from breast cancer were included in this retrospective study. Of these patients, 30 were treated with radiosurgery alone and 28 with radiosurgery plus whole-brain radiotherapy. Both groups were compared for local control of the irradiated metastases, freedom from new brain metastases and survival. Furthermore, eight additional factors were analyzed including dose of radiosurgery, age at radiotherapy, Eastern Cooperative Oncology Group (ECOG) performance score, number of brain metastases, maximum diameter of all brain metastases, site of brain metastases, extra-cranial metastases and the time from breast cancer diagnosis to radiotherapy.

**Results:**

The treatment regimen had no significant impact on local control in the univariate analysis (*p* = 0.59). Age ≤59 years showed a trend towards improved local control on univariate (p = 0.066) and multivariate analysis (*p* = 0.07). On univariate analysis, radiosurgery plus whole-brain radiotherapy (*p* = 0.040) and ECOG 0–1 (*p* = 0.012) showed positive associations with freedom from new brain metastases. Both treatment regimen (*p* = 0.039) and performance status (*p* = 0.028) maintained significance on multivariate analysis. ECOG 0–1 was positively correlated with survival on univariate analysis (p < 0.001); age ≤59 years showed a strong trend (*p* = 0.054). On multivariate analysis, performance status (*p* < 0.001) and age (*p* = 0.041) were significant.

**Conclusions:**

In breast cancer patients with few brain metastases, radiosurgery plus whole-brain radiotherapy resulted in significantly better freedom from new brain metastases than radiosurgery alone. However, this advantage did not lead to significantly better survival.

## Background

In general, the prognosis of patients with brain metastasis from breast cancer is more favorable than the prognosis of patients with other primary tumors such as non-small lung cancer [1-3]. Therefore, breast cancer patients developing brain metastases are a group of cancer patients requiring particular attention. About 50% of breast cancer patients developing brain metastases have more than three lesions and are mostly candidates for whole-brain radiotherapy alone [2].

Patients with one to three brain metastases have a much more favorable survival prognosis and may, therefore, benefit from more intensive therapies including surgical resection and radiosurgery. In the majority of these patients, radiosurgery is performed, either alone or supplemented by whole-brain radiotherapy [4]. However, the question whether the addition of whole-brain radiotherapy is beneficial or harmful, still needs to be answered properly. Three randomized trials demonstrated that additional whole-brain radiotherapy leads to better local control and freedom from new brain metastases without extending survival [5-7]. However, these trials included patients with brain metastases from different primary tumors. Because of the specific biology and clinical course of each tumor entity, the value of adding whole-brain radiotherapy to radiosurgery for a limited number of brain metastases may vary. Sperduto et al. identified different prognostic factors of survival for each common primary tumor associated with brain metastases including lung cancer, renal cell carcinoma, melanoma, gastrointestinal cancer and breast cancer [8]. Therefore, it appears reasonable to take a separate look at each tumor entity. Breast cancer is the second most common primary tumor in patients presenting with brain metastases. In contrast to the three randomized trials including various primary tumors [5-7], a retrospective study focusing on patients treated with Gamma Knife radiosurgery for brain metastases from breast cancer suggested no difference in local control and freedom from new brain metastases with the addition of whole-brain radiotherapy [9]. Thus, further studies are required to better define the role of additional whole-brain radiotherapy in breast cancer patients. Therefore, the present study was initiated. It compared radiosurgery alone to radiosurgery supplemented by whole-brain radiotherapy in patients with 1–3 brain metastases from breast cancer treated with linear accelerator based radiosurgery or CyberKnife radiosurgery.

## Patients and methods

In this retrospective study, the data of 30 breast cancer patients receiving radiosurgery alone for 1–3 newly diagnosed brain metastases without leptomeningeal spread were compared to the data of 28 patients who had received radiosurgery supplemented by whole-brain radiotherapy for the same indication. Patients did not have prior radiosurgery or whole-brain radiotherapy. Both groups were compared for local control of the treated lesions, freedom from new brain metastases and survival. The patients had received radiosurgery with either linear accelerator (n = 51) or CyberKnife (n = 7). The dose was prescribed to the margin of the metastases, representing the 75-90% isodose level.

Eight additional variables were evaluated for treatment outcomes including the dose of radiosurgery (<20 Gy versus ≥20 Gy, prescribed to the margin of the metastases, representing the 75-90% isodose level), age (≤59 versus ≥60 years), Eastern Cooperative Oncology Group (ECOG) performance score (ECOG 0–1 versus ECOG 2), number of brain metastases (1 versus 2–3), maximum diameter of all brain metastases (≤10 versus ≥11 mm, median diameter: 11 mm), site of brain metastases (supratentorial versus infratentorial ± supratentorial), extra-cranial metastases (no versus yes) and time from breast cancer diagnosis to radiotherapy (≤48 versus ≥49 months). These variables were similarly distributed in the two treatment groups (Table 1). This retrospective study was approved by the ethics committee of the University of Lübeck.

**Table 1:** **Characteristics of the treatment groups**

	**Radiosurgery alone N patients (%)**	**Radiosurgery + WBRT N patients (%)**	***P*** **-value**
**Radiosurgery dose**			
<20 Gy (n = 23)	10 (33)	13 (46)	
≥20 Gy (n = 35)	20 (67)	15 (54)	0.63
**Age**			
≤59 years (n = 34)	17 (57)	17 (61)	
≥60 years (n = 24)	13 (43)	11 (39)	0.94
**ECOG performance score**			
0-1 (n = 40)	20 (67)	20 (71)	
2 (n = 18)	10 (33)	8 (29)	0.93
**Number of cerebral metastases**			
1 (n = 46)	25 (83)	21 (75)	
2-3 (n = 12)	5 (17)	7 (25)	0.84
**Maximum diameter of all brain metastases**			
≤10 mm (n = 28)	15 (50)	13 (46)	
≥11 mm (n = 30)	15 (50)	15 (54)	0.95
**Site of brain metastases**			
Supratentorial (n = 39)	20 (67)	19 (68)	
Infratentorial ± supratentorial (n = 19)	10 (33)	9 (32)	0.98
**Extra-cranial metastases**			
No (n = 24)	11 (37)	13 (46)	
Yes (n = 34)	19 (63)	15 (54)	0.75
**Time from breast cancer diagnosis to radiotherapy**			
≤48 months (n = 30)	15 (50)	15 (54)	
≥49 months (n = 28)	15 (50)	13 (46)	0.95

*P*-values were obtained with the Chi-square test.

Local control, freedom from new brain metastases and survival were measured from the end of radiotherapy. Follow-up was generally performed with magnetic resonance imaging about six weeks and about every three to four months following irradiation, and additionally in case of new or progressive symptoms. Local failure was defined as increase of the diameter of the treated lesions by at least 25%. In such a situation, a specialized neuro-radiologist was consulted to allow the differentiation between progression and pseudo-progression. Distant intracerebral failure (new brain metastases) did not include leptomeningeal spread.

The univariate analyses of local control, freedom from new brain metastases and survival were performed with the Kaplan-Meier analysis and the log-rank test. In the radiosurgery alone group, six patients received whole-brain radiotherapy and six patients fractionated stereotactic radiotherapy for either local or distant intracerebral failure during the further course of disease. In the radiosurgery plus whole-brain radiotherapy group, four patients received radiosurgery or fractionated stereotactic radiotherapy for new brain metastases. These patients were censored for local control and freedom from new brain metastases at the time when local progression or new brain metastases were detected.

Variables achieving significance or showing a trend on univariate analyses (*p <* 0.07) were subsequently evaluated with the Cox regression analysis (multivariate analysis). Median follow up was 13 months (range: 1–39 months) in the entire series and 14 months (range: 12–39 months) in the patients alive at the last follow up.

## Results

Younger age (≤59 years) showed a trend towards improved local control of the irradiated metastases on both univariate analysis (*p* = 0.066) and subsequent multivariate analysis (risk ratio (RR): 2.99; 95%-confidence interval (CI): 0.90-11.42; *p* = 0.07). The treatment regimen had no significant impact on local control in the univariate analysis (*p* = 0.59). The results of the whole univariate analysis of local control are presented in Table 2.

**Table 2:** **Local control of the irradiated brain metastases (univariate analysis)**

	**At 6 months (%)**	**At 12 months (%)**	***P*** **-value**
**Treatment regimen**			
Radiosurgery alone (n = 30)	86	79	
Radiosurgery + WBI (n = 28)	96	92	0.59
**Radiosurgery dose**			
<20 Gy (n = 23)	83	78	
≥20 Gy (n = 35)	97	90	0.23
**Age**			
≤59 years (n = 34)	97	91	
≥60 years (n = 24)	83	77	0.066
**ECOG performance score**			
0-1 (n = 40)	95	87	
2 (n = 18)	82	82	0.69
**Number of cerebral metastases**			
1 (n = 46)	91	83	
2-3 (n = 12)	92	92	0.58
**Maximum diameter of all brain metastases**			
≤10 mm (n = 28)	93	89	
≥11 mm (n = 30)	90	81	0.66
**Site of brain metastases**			
Supratentorial (n = 39)	92	83	
Infratentorial ± supratentorial (n = 19)	89	89	0.35
**Extra-cranial metastases**			
No (n = 24)	88	79	
Yes (n = 34)	94	91	0.31
**Time from breast cancer diagnosis to radiotherapy**			
≤48 months (n = 30)	93	86	
≥49 months (n = 28)	89	85	0.61

**Figure 1:**
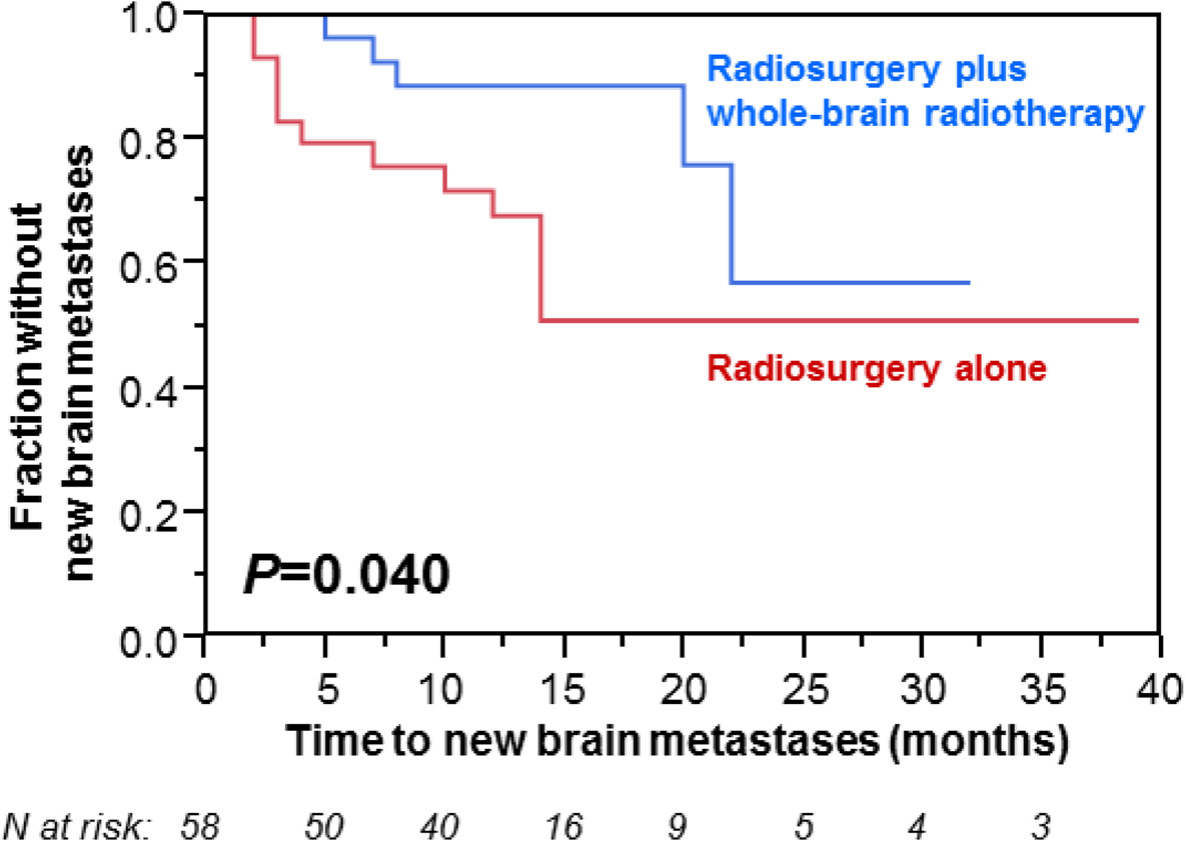
**Kaplan-Meier analysis of freedom from new brain metastases: Radiosurgery** ***vs.*** **radiosurgery plus whole-brain radiotherapy.**

On univariate analyses of freedom from new brain metastases, radiosurgery supplemented by whole-brain radiotherapy (*p* = 0.040, Figure 1) and an ECOG performance score of 0–1 (*p* = 0.012) showed a positive association with this endpoint (Table 3). In the corresponding Cox regression analysis, the treatment regimen (RR: 2.95; 95%-CI: 1.05-9.52; *p* = 0.039) and the performance status (RR: 3.44; 95%-CI: 1.15-10.10; *p* = 0.028) maintained significance.

**Table 3:** **Freedom from new brain metastases (univariate analysis)**

	**At 6 months (%)**	**At 12 months (%)**	***P*** **-value**
**Treatment regimen**			
Radiosurgery alone (n = 30)	79	68	
Radiosurgery + WBI (n = 28)	96	89	**0.040**
**Radiosurgery dose**			
<20 Gy (n = 23)	78	74	
≥20 Gy (n = 35)	94	81	0.72
**Age**			
≤59 years (n = 34)	91	88	
≥60 years (n = 24)	83	61	0.20
**ECOG performance score**			
0-1 (n = 40)	90	82	
2 (n = 18)	82	66	**0.012**
**Number of cerebral metastases**			
1 (n = 46)	87	74	
2-3 (n = 12)	92	92	0.98
**Maximum diameter of all brain metastases**			
≤10 mm (n = 28)	89	81	
≥11 mm (n = 30)	86	74	0.21
**Site of brain metastases**			
Supratentorial (n = 39)	89	75	
Infratentorial ± supratentorial (n = 19)	84	84	0.50
**Extra-cranial metastases**			
No (n = 24)	92	73	
Yes (n = 34)	85	82	0.89
**Time from breast cancer diagnosis to radiotherapy**			
≤48 months (n = 30)	90	82	
≥49 months (n = 28)	86	73	0.33

Significant p-values are given in bold.

An ECOG performance score of 0–1 was positively correlated with survival in the corresponding univariate analysis (*p* < 0.001). Furthermore, younger age (≤59 years) showed a strong trend (*p* = 0.054) towards better survival (Table 4). In the following Cox regression analysis, the performance score (RR: 5.05; 95%-CI: 2.35-11.02; *p* < 0.001) and age (RR: 2.22; 95%-CI: 1.04-4.84; *p* = 0.041) were significant.

**Table 4:** **Survival (univariate analysis)**

	**At 6 months**	**At 12 months**	***P*** **-value**
**Treatment regimen**			
Radiosurgery alone (n = 30)	80	63	
Radiosurgery + WBI (n = 28)	93	71	0.48
**Radiosurgery dose**			
<20 Gy (n = 23)	83	65	
≥20 Gy (n = 35)	89	69	0.56
**Age**			
≤59 years (n = 34)	91	76	
≥60 years (n = 24)	79	54	0.054
**ECOG performance score**			
0-1 (n = 40)	98	85	
2 (n = 18)	61	28	**<0.001**
**Number of cerebral metastases**			
1 (n = 46)	83	63	
2-3 (n = 12)	100	83	0.67
**Maximum diameter of all brain metastases**			
≤10 mm (n = 28)	89	79	
≥11 mm (n = 30)	83	57	0.16
**Site of brain metastases**			
Supratentorial (n = 39)	85	64	
Infratentorial ± supratentorial (n = 19)	89	74	0.22
**Extra-cranial metastases**			
No (n = 24)	88	75	
Yes (n = 34)	85	62	0.38

Significant p-values are given in bold.

## Discussion

Breast cancer patients account for 20-25% of all cancer patients developing brain metastases [1].

The majority of breast cancer patients with a very limited number of brain metastases have a relatively favorable prognosis and are, therefore, candidates for intensive local therapies, mostly radiosurgery [10].

It is not clear whether the results of radiosurgery can be further improved with the addition of whole-brain radiotherapy. In a previous retrospective study of 62 breast cancer patients receiving Gamma Knife radiosurgery, additional whole-brain radiotherapy did not result in better local control, freedom from new brain metastases and survival [9]. However, those findings contrast with the results of two randomized trials comparing radiosurgery alone to radiosurgery supplemented by whole-brain radiotherapy in patients with very few brain metastases from different primary tumors [6,7]. The trial of Kocher et al. compared local treatment (radiosurgery or neurosurgical resection) alone to local treatment plus whole-brain radiotherapy for 1–3 brain metastases [6]. In the 199 patients receiving radiosurgery, the addition of whole-brain radiotherapy resulted in improved local control of the treated brain lesions (31% *vs.* 19%, *p* = 0.040) and improved freedom from new brain metastases (48% *vs.* 33%, *p* = 0.023) after two years. Survival was similar in both groups (*p* = 0.24). Another randomized trial from Japan compared radiosurgery alone to radiosurgery plus whole-brain radiotherapy in 132 patients with 1–4 brain metastases from different primaries [7]. The combined approach led to better local control (89% *vs.* 73%, *p* = 0.002) and freedom from new brain metastases (58% *vs.* 36%, *p* = 0.003) at one year. One-year survival rates were 39% and 28%, respectively (*p* = 0.42). The inconsistency of the findings of the retrospective study of breast cancer patients receiving Gamma Knife radiosurgery and the two randomized trials may be explained by the study design, the different distribution of primary tumors and by the fact that the retrospective study had included also patients with more than very few brain metastases [6,7,9]. The contradictory results demonstrate that the value of additional whole-brain radiotherapy for very few brain metastases from breast cancer needs further clarification. Therefore, the present study was initiated. In contrast to the preceding retrospective study focusing on breast cancer patients, radiosurgery in our present study was performed with a linear accelerator and not with a Gamma Knife [9]. Furthermore, the number of brain metastases did not exceed three lesions in our study. According to the results of our study, additional whole-brain radiotherapy improved freedom from new brain metastases but not survival. These findings mostly agree with the results of the two randomized trials of patients with different primary tumors [6,7]. This means that for breast cancer patients with very few brain metastases, the decision whether or not to add whole-brain radiotherapy is difficult. The authors of a randomized trial of 58 patients suggested a more pronounced decline in neurocognitive function at four months if the combined approach was given and emphasized radiosurgery alone [5]. In contradiction, another prospective study of 92 patients, who had been previously included in a randomized trial of 132 patients, suggested decline in neurocognitive function to a lesser extent after radiosurgery plus whole-brain radiotherapy than after radiosurgery alone both at one and at two years following treatment [11]. The better neurocognitive function in the radiosurgery plus whole-brain radiotherapy group was explained by the significantly better intra-cerebral control achieved with the addition of whole-brain radiotherapy. Authors of two other prospective trials also stated that an intra-cerebral recurrence is the most important cause of neurocognitive decline in patients with metastasis to the brain [12,13]. However, all of these prospective studies included patients with brain metastases from many different primary tumor types.

In order to facilitate the decision for or against the addition of whole-brain radiotherapy, a predictive instrument would be helpful that estimates the risk of new brain metastases. Such an instrument is already available [14]. However, it was also created from patients with brain metastases from different primaries. A specific instrument for breast cancer patients would be even better for predicting the risk of new brain metastases as precisely as possible but will likely not be available in the near future.

In addition to the impact of the treatment regimen on freedom from new brain metastases, a better performance status was significantly associated with freedom from new brain metastases and survival, and younger age showed a strong trend towards improved survival in the present study. These findings agree with the results of previous studies. The performance status showed significant associations with survival in two retrospective studies from Korea, performed in 62 and106 breast cancer patients, respectively, treated with Gamma Knife radiosurgery [9,15]. In another retrospective study of 100 patients from the United States who were treated with Gamma Knife radiosurgery for brain metastasis from breast cancer, age was a strong predictor of survival [16]. These data suggest some consistency of the results of the current study despite its limitations due to the retrospective design.

In summary, freedom from new brain metastases was significantly improved with the addition of whole-brain irradiation to radiosurgery in breast cancer patients with 1–3 brain metastases. Unfortunately, better freedom from new brain metastases was not associated with improved survival. Further studies are required to properly identify those patients who clearly benefit from the addition of whole-brain radiotherapy to radiosurgery.
